# Supplementary material for: Empirical methods for controlling false positives and estimating confidence in ChIP-Seq peaks
Source: BMC Bioinformatics. 2008 Dec 5;9:523. doi: 10.1186/1471-2105-9-523 (PMC2628906; doi:10.1186/1471-2105-9-523)
Supplement: Additional File 1 — A variety of html documents from the USeq web site detailing the available applications, their best usage, output file type descriptions, command line menus, etc. [file 1471-2105-9-523-S1.zip › USeqUserGuides/JavaDoc.zip!/JavaDoc/trans/graphics/package-frame.html]

trans.graphics


trans.graphics

Classes 
  
AggregatePlots
  
Box
  
CelMasker
  
CombineXYSeries
  
GenomicRegion
  
GenomicRegionGlyph
  
Graph
  
IntervalDrawFrame
  
IntervalDrawPanel
  
IntervalPlotter
  
PlotFloatArrays
  
RankedSetAnalysis
  
RankedSetDrawFrame
  
RankedSetDrawPanel
  
VirtualCel
  
VirtualCelFrame
  
VirtualCelPanel
